# A Retrospective Two-Year Review of the Outcomes of Surgical Rib Fixation Following Chest Wall Injury by the Multidisciplinary Chest Wall Injury Group in a Major Trauma Centre and the Change in Outcomes as the Service Has Developed

**DOI:** 10.7759/cureus.44950

**Published:** 2023-09-09

**Authors:** Abbas Shahid, Thomas Turner, Ali Bukhari, Adil Shaikh, Asad Malik, Hatim Alsusa, Kieran Bowdren, Jill Rutherford

**Affiliations:** 1 Orthopaedics, Manchester Medical School, University of Manchester, Manchester, GBR; 2 Emergency Medicine, University Hospitals of North Midlands NHS Trust, Manchester, GBR; 3 Vascular Surgery, Northern Care Alliance NHS Group, Manchester, GBR; 4 Emergency Medicine, Warrington and Halton Hospitals NHS Trust, Manchester, GBR; 5 Vascular Surgery, Manchester Medical School, University of Manchester, Manchester, GBR; 6 Trauma and Orthopaedics, Salford Royal NHS Foundation Trust, Manchester, GBR

**Keywords:** surgical stabilisation of rib fractures, orthopaedic trauma, rib fixation, chest trauma score, incidence of mortality, chest wall trauma, surgical fixation, flail chest, multiple rib fractures

## Abstract

Aims

All English major trauma centres (MTCs) offer rib fixation, which the National Institute for Health and Care Excellence (NICE) guidance indicates in patients with multiple rib fractures or a flail segment; however, the data does not identify the appropriate patients. Our aims were to establish improvements in outcomes following rib fixation at our trust and then determine if the rib fixation service has improved.

Methods

We performed a matched cohort study whereby 32 patients who underwent rib fixation were independently matched with conservatively managed patients. We then performed a retrospective re-audit to compare outcomes with the matched cohort study. The outcomes analysed were mortality, critical care length of stay (LOS) and total hospital LOS.

Results

Our initial study revealed a 33.4% reduction in mortality in patients over 55 years. There was also a reduction in average total hospital LOS by 4.5 days in patients under 55 years when comparing rib fixation to conservative management. The results also revealed an average of 4.1 days from admission to operation, 12.7 days of critical care LOS and 29.1 days of total hospital LOS. The re-audit showed improvements in all outcomes. Time from admission to fixation was reduced to 2.1 days, critical care LOS was reduced to 7.5 days and total hospital LOS was reduced to 20.7 days.

Conclusions

Reduced mortality and LOS reinforce evidence that rib fixation improves outcomes. The re-audit shows that patients are identified for fixation sooner, which is important as the evidence has not identified optimal time for fixation. LOS further decreased in our re-audit, which indicates that earlier fixation results in patients avoiding the sequelae of rib fractures.

## Introduction

Background

Trauma is a major cause of morbidity, and worldwide, it is the single biggest cause of death in those under the age of 40 [1]. Approximately one in five traumatic deaths can be attributed to high-energy blunt thoracic trauma and its sequelae alone [2,3].

Chest wall injuries are common and range from minor bruising to life-threatening crush injuries [4]. Rib fractures can be seen in as many as 10% of patients who are subjected to trauma [5,6] and approximately 25%-30% in the case of significant thoracic trauma [7,8]. The presence of rib fractures increases the risk of thoracic complications associated with organ injury and altered ventilation [9], including pneumonia and atelectasis. The majority (75%) of trauma-related rib fractures can be attributed to blunt trauma, with road traffic collisions (RTCs) being the biggest contributor. The remaining 25% occurs as a result of penetrating injuries to the chest [10].

The management strategy for rib fractures globally is largely conservative [3,7]. All major trauma centres (MTCs) offer rib stabilisation when deemed clinically appropriate using plates and screws to stabilise the fractured segment of the rib with simple fractures managed conservatively [3,7]. The most recent guidance published by the National Institute for Health and Care Excellence (NICE) in October 2010 [4] indicates that chest wall stabilisation using a surgical approach is beneficial in patients with multiple rib fractures or a flail segment. The benefit is in terms of improved lung function and reduction in both critical care length of stay (LOS) and overall hospital LOS. The surgical stabilisation of rib fractures in the appropriate patients has revealed encouraging initial results. Whilst the majority of patients subjected to rib fractures will regain their baseline respiratory function and be able to manage the associated pain without the surgical stabilisation of their rib fractures, it is thought that there are specific groups of patients who would physiologically benefit from stabilisation [8]. MTCs across the United Kingdom have implemented local guidelines with criteria to try to identify the specific groups of patients; however, further data is needed to be able to configure national standards and a guideline for the management of rib fractures.

Each year, hundreds of patients attend Salford Royal NHS Foundation Trust (SRFT) following trauma. Approximately 12% of these patients will undergo surgical rib fixation based on specific trust criteria and clinical judgement. By reviewing the hospital records of patients who underwent operative chest wall stabilisation, this project aims to identify the current trends and overall outcomes in rib fixation at SRFT and thus determine the efficacy of the rib fixation service as it currently functions.

Morbidity and mortality of rib fractures

The most common ribs fractured in trauma are ribs four to nine [7,11]. The type of fracture can vary from simple un-displaced fractures in isolated ribs that rarely cause any clinical difficulties to complex crush fractures with the potential for serious pulmonary complications [8]. The severity of damage inflicted to the skeletal structures of the thorax is often an accurate reflection of the force sustained at the time of injury; for example, a fracture to the first rib is rare and requires a significant amount of energy [12]. In patients with poor bone physiology, rib fractures can occur with lower-energy injuries, for example, in the elderly population or those with established osteoporosis or osteopenia [8]. Furthermore, studies show that with each additional rib fracture sustained, the patient’s risk of morbidity and mortality rises [8,11].

The morbidity and mortality that are associated with rib fractures are a result of one or a combination of the following: reduced ventilation secondary to pain at the site of injury, inability to cough and evacuate pulmonary secretions, tissue damage to lung parenchyma under the fracture site that impairs gaseous exchange and the disruption of the chest wall anatomy causing alterations in breathing mechanics, particularly in the presence of a flail chest segment [1,7,13]. These factors either cause concurrent injuries such as major vessel injury (e.g. aortic dissection), haemothorax, pneumothorax and other pulmonary contusions or predispose the patient to pulmonary complications such as pneumonia, atelectasis, acute respiratory distress syndrome (ARDS) and respiratory failure, which necessitates mechanical ventilation [14]. Respiratory complications are the leading cause of morbidity associated with rib fractures, with pneumonia being the most commonly seen in up to 30% of patients [8].

An indication for surgical chest wall stabilisation is a chest with a flail segment, also known as a flail chest. By definition, a flail segment occurs when two or more fractures occur per rib in at least two adjacent ribs, and the separation of this segment from the chest wall produces a flail chest [3]. This produces characteristic paradoxical movements of the segment during normal ventilation, whereby the flail segment moves inwards on inspiration [1]. As a consequence, the lung beneath this segment of flail chest does not contribute to ventilation, and so, oxygen requirements are often much higher in patients suffering this injury [3]. Often, these patients and those with non-flail multiple displaced rib fractures will require additional ventilatory support with positive pressure ventilation to counteract or prevent respiratory failure [3,7]. Both morbidity and mortality are higher in patients with a flail chest, with mortality as high as 33% [1,13,14].

Surgical fixation of the ribs

The approach to the management of rib fractures has varied over the past few decades; until recently, the conservative management of rib fractures was the norm. Developments in critical care medicine have furthered support and provision for a non-operative management. However, evidence from the international community has developed a change in trend to surgical chest wall stabilisation.

Chest wall stabilisation devices were developed historically, and more recently, there has been further development of old techniques and the introduction of new rib fixation devices and materials. There is also growing evidence in this area that has led to international recognition that rib fixation has a place in the management of rib fractures [8]. The evidence suggests that certain groups of patients may benefit from fixation, in particular those with flail chests, multiple rib fractures, deteriorating respiratory function and respiratory failure [1].

The main goal of open reduction and internal fixation (ORIF) is to reduce pain in a timely manner and restore the normal respiratory mechanics of the chest wall whilst optimising lung function to prevent the deterioration of a patient [12]. The movement of the chest wall is painful when rib fractures are present, and so, the adequate control of pain itself enhances the patient’s ventilatory function; also, the stabilisation of the chest wall can improve ventilatory function [11] by improving the mechanics of breathing. Earlier fixation is believed to avoid some of the sequelae associated with rib fractures, such as pneumonia, and hence improve patient outcomes [15].

The NICE guidance [4] comments on the evidence for the insertion of metal rib reinforcements to stabilise a flail chest wall as being ‘limited in quantity but consistently shows efficacy’. It is also recommended that patients should be selected by the appropriate specialists, namely, critical care specialists, chest physicians and thoracic surgeons [4]. This evidence is based on a single randomised controlled trial (RCT) and one comparative study. In 2016, a meta-analysis of three relatively small RCTs (123 patients in total) concluded that the surgical approach to rib fractures was associated with significant clinical benefits when comparing 123 patients who underwent surgical stabilisation to nonsurgical management. This was seen in the reduction in the incidence of pulmonary complications (the incidence of pneumonia reduced by two-thirds), length of stay in intensive care and total length of stay in the hospital [3]. The quality of life following rib fixation was investigated by Mayberry et al. [16], who concluded that there was low morbidity in the long term, as well as little or no pain.

Today, all MTCs are required to offer a rib fixation service as determined by the NHS England trauma peer review of 2015. This has seen a big movement towards surgical chest wall stabilisation and to define more precise indications for fixation [8].

SRFT rib fixation protocol

SRFT implemented a protocol for the management of rib fractures [12]. It was developed by the SRFT Chest Injuries Group, which is composed of clinicians including pain management anaesthetic specialists, trauma and orthopaedic surgeons, intensivists, orthogeriatricians, respiratory therapists, pain nurses and trauma coordinators. The group formed in response to the National Trauma Review of 2015 [17]. The clinicians at SRFT recognised that the role of rib fixation is not truly understood, and so, the optimal decision of how to manage rib fracture patients would be most beneficial via a multidisciplinary approach. National rib fixation has been undertaken by three specialties depending on how the service has developed locally. In SRFT, orthopaedic surgeons undertake the surgery, but in other centres, it is undertaken by cardiothoracic or general surgeons.

Rib fractures and underlying pulmonary contusions are poorly identified radiographically. The surgeons use computer tomography (CT) with three-dimensional (3D) reconstructions to plan their fixation.

Ribs four to nine provide the greatest stability to the chest wall, and ribs one and two are difficult to access; therefore, only those fractures between ribs three and 10 (inclusive) are fixed [7]. The number of ribs that require fixation in order to achieve satisfactory stabilisation is not yet known. However, as rib fixation is merely a stabilisation technique and absolute stability is not required, it is thought that it is not necessary to fix every fractured rib. At SRFT, the surgeons fix alternate ribs and then test if the stability of the chest wall has been re-established. If stabilisation is not satisfactory, they will fix the ribs between those already fixed. The DePuy MatrixRIB™ Fixation System is currently used at SRFT.

Aims and objectives

Since September 2015, a rib fixation service was established at SRFT, an MTC in the north-west of England. Over this period up until 25 May 2018 a total of 50 patients had undergone chest wall stabilisation for multiple rib fractures. Our first aim was to establish if there was any improvement in patient outcomes following rib fixation. We compared the first 32 patients who had rib fixation to a matched group of SRFT patients before rib fixation was undertaken. Our second aim was to determine if the rib fixation service has modified its processes by comparing the first 32 patients with the following patients. The outcomes we looked at were mortality, length of stay in critical care and total hospital stay. As there is little literature surrounding rib fixation, the questions we were trying to answer are, does operative management improve outcomes compared to non-operative management and what are the indications for fixation and in what time period?

## Materials and methods

Selection

Initial pain management strategies and the level of care escalation from the emergency department (ED) are guided by the rib fracture score (RFS) [18], which is calculated on admission using the formula outlined in Table 1. It is recognised that many patients with rib fractures can deteriorate following admission, and so, the RFS is a means of quantifying the severity of injury [12]. This identifies those patients who are more likely to have respiratory sequelae and pain management issues as a result of their fractures.

**Table 1:** Values provided for each category Formula used to calculate rib fracture score: RFS = (Breaks x Sides) + Age Factor Score [13,19]

Breaks	Sides	Age Factor Score
Number of fractured ribs in total	Unilateral = 1	<50 = 1
		51-60 = 2
	Bilateral = 2	61-70 = 3
		71-80 = 4
		>80 = 5

Within the SRFT protocol is guidance as to which patients should be considered for surgical management [12]. These criteria should be evaluated by a combination of clinical assessment and investigative techniques by the appropriately qualified clinician managing the patient in question. The specific criteria that clinicians should be aware of when considering referral for ORIF are detailed in Table 2. Following admission to the ED, all patients with rib fractures are assessed by the pain team. If pain management regimes are not effective and the patient’s respiratory function deteriorates, they are often identified as a candidate for fixation.

**Table 2:** Patient demographics and clinical characteristics of both the non-operative and rib fixation groups of patients NS: no significance

		Group				P-Value
		Non-operative		Rib fixation		
		Mean	Count	Mean	Count	
Age		59.77		59.40		NS (0.976)
Sex	Female		13		13	NS (1.000)
	Male		17		17	
Co-morbidities	Minor		14		10	NS (0.292)
	Major		16		20	
Number of ribs fractured		N = 8		N = 9		NS (0.237)
Type of fractures	Unilateral, no flail		5		6	No statistical test appropriate
	Unilateral with flail		15		9	
	Bilateral, no flail		6		5	
	Bilateral with flail		4		10	
Mechanism of injury	Vehicle versus vehicle		1		2	No statistical test appropriate
	Pedestrian versus vehicle		12		11	
	Blunt trauma, car rolled on top		1		1	
	Fall of less than 2 m		8		8	
	Fall greater than 2 m		8		8	

Patients are also considered for surgical rib fixation if they possess three or more displaced rib fractures or a flail segment, alongside any one of the following: pain from the injured chest wall despite optimal analgesia, poor ventilation with high oxygen requirements, pre-existing chest co-morbidities (chronic obstructive pulmonary disease {COPD}, asthma and bronchiectasis) or pre-existing conditions contraindicating epidural analgesia.

Matched cohort study (2017)

In 2017, we performed a matched cohort study whereby patients who underwent rib fixation between 1 September 2015 and 27 March 2017 were independently matched with patients who, prior to 1 September 2015, were managed conservatively. The dates of the study period signify the date in which the fixation service began at SRFT and the latest date whereby data could have been obtained, respectively.

The variables that we matched to formulate the conservatively managed group included age, sex, the mechanism of injury, number of ribs fractured and the type of fracture (i.e. flail or non-flail and whether it was unilateral or bilateral). We also matched patient co-morbidities categorising them as ‘minor’ or ‘major’ depending on how significantly the co-morbidity affects health.

Patients were classified as having minor conditions if they were fit and well and have asthma, atrial fibrillation, hypertension, hypothyroidism, osteoporosis, rheumatoid arthritis, epilepsy or dementia. Patients were classified as having major co-morbidities if they have a history of COPD, myocardial infarction, heart failure, cerebrovascular accident, renal impairment, diabetes, mental health condition (schizophrenia/bipolar) or alcohol abuse.

We analysed specific patient demographics and clinical characteristics of both the surgically managed group and the conservative group, which we then compared. The data analysed included the following: patient demographics and co-morbidities, mechanism of injury, number of ribs fractured and whether there was an associated flail segment and number of ribs fixed. We also analysed specific outcome data to determine whether the surgical management of patients with rib fractures improved outcomes when compared to conservatively managed patients.

Service evaluation (2018)

In May 2018, we performed a retrospective re-audit to compare outcomes since our matched cohort study in 2017. To do so, we identified all patients who underwent rib fixation between 28 March 2017 and 25 May 2018. The data collected in this study follows on from the last patient in the previous study to ensure all patients who have ever undergone rib fixation at SRFT are included. This gave us a new cohort of surgically managed patients for which to compare outcomes with patients who underwent rib fixation in our matched cohort study in 2017.

We analysed the same patient demographics and clinical characteristics of this new cohort of patients, including patient demographics and co-morbidities (see Table 3), mechanism of injury, number of ribs fractured and whether there was an associated flail segment and number of ribs fixed. This was then compared with the cohort of surgically managed patients of our matched cohort study of 2017.

**Table 3:** Comparison of death between the non-operative and rib fixation groups of patients

		Group		Total
		Non-operative	Rib fixation	
Complication of death	No	19	29	48
	Yes	11	1	12
Total		30	30	60

Outcome analysis

Using the two groups of the matched cohort study in 2017 and the service evaluation of 2018, we analysed and compared outcome data of both surgically managed cohorts of patients. To do so, we divided each cohort into two distinct groups based on age: the under 55 and over 55 groups.

We then compared the results of the two separate studies, and this allowed us to establish whether the Chest Injuries Group have modified their approach to the management of rib fracture patients as the service has developed and if that has altered their results and subsequently helped them in identifying those patients who would benefit from fixation.

The outcomes we used in our analysis were as follows: mortality rate, time from emergency admission to the patient’s rib fixation operation (in days), critical care LOS (in days) and overall hospital LOS (in days).

Statistical tests

When analysing this data, we used two primary statistical tests. For comparing continuous variable, we used the Mann-Whitney test, and for categorical variables, we used the chi-square test. We set the significance level with a p-value of less than 0.05 for both tests. To work out all the scores and dates required for the outcome measures, we collected patient data from the electronic patients record (EPR) system at SRFT.

## Results

Matched cohort study (2017)

The key variables we matched each patient on are demonstrated in Table 2, alongside the numbers in each category. There was no significant statistical difference between the rib fixation group and the conservatively matched group in any of the matched variables.

Eleven patients died in the conservatively managed group as compared to one in the rib fixation group as shown in Table 3. This presented a reduction in mortality from 36.7% to 3.3.%, which was proved to be statistically significant with a p-value of 0.001. Of the patients that died in the conservative group, nine (81.8%) died within the first 28 days, with two (18.2%) passing away three months after the incident. This is in stark comparison to the operative group where there were no immediate or significantly late deaths, with only one person dying after 28 days.

**Figure 1:**
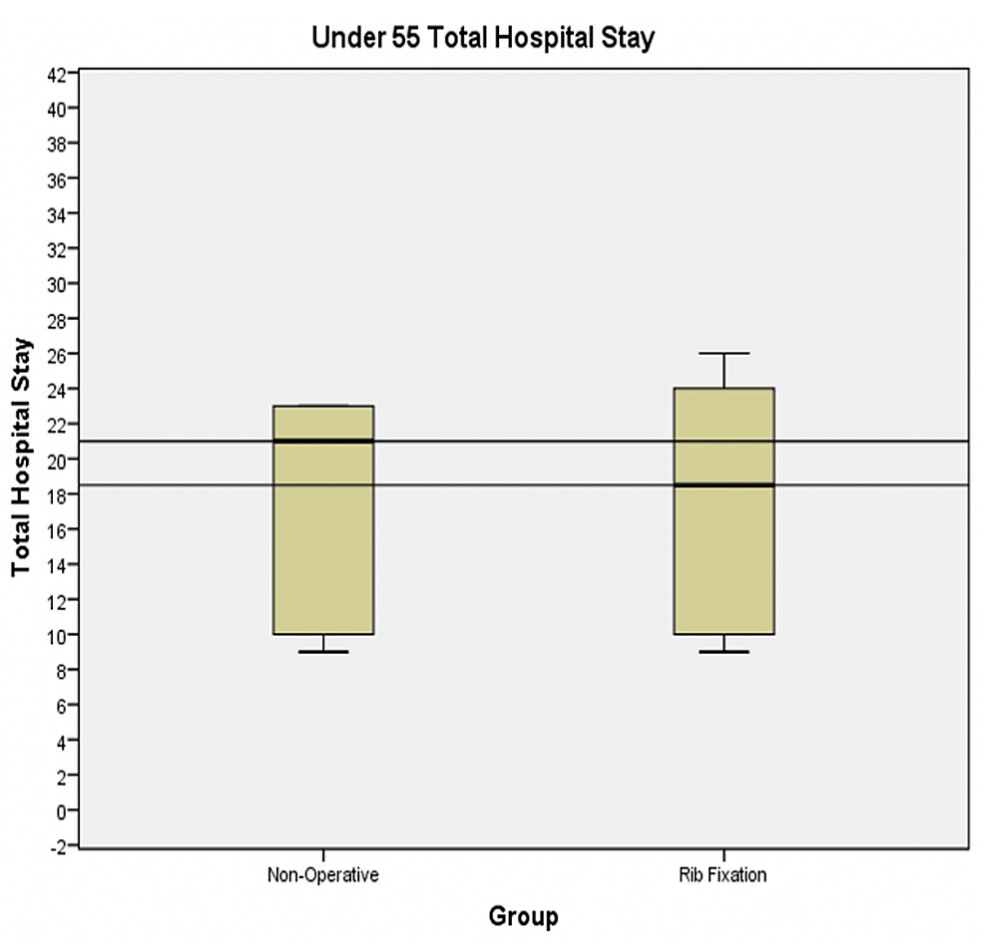
Box plots showing the total length of stay in the non-operatively managed and rib fixation cohorts for all patients <55 years old

For the under 55 subgroup, the mean total length of stay was 22.13 days in the non-operative group compared to 17.60 days in the operative group, showing a reduction in length of stay of 4.53 days. The median includes all the data, with the 753-day total length of stay of one patient excluded. The median was 21 days in the non-operative group and 18.50 days in the operative group. This highlights a reduction of 2.5 days, which is visually demonstrated in Figure 1. The patient with a 753-day total length of stay value was excluded because this patient had an excessively long stay in the neuro-rehabilitation unit. In a typical hospital setting, this patient would have been discharged to intermediate health care, but as this service was offered at SRFT (being a specialist neurology centre), this patient remained on this rehabilitation ward.

**Figure 2:**
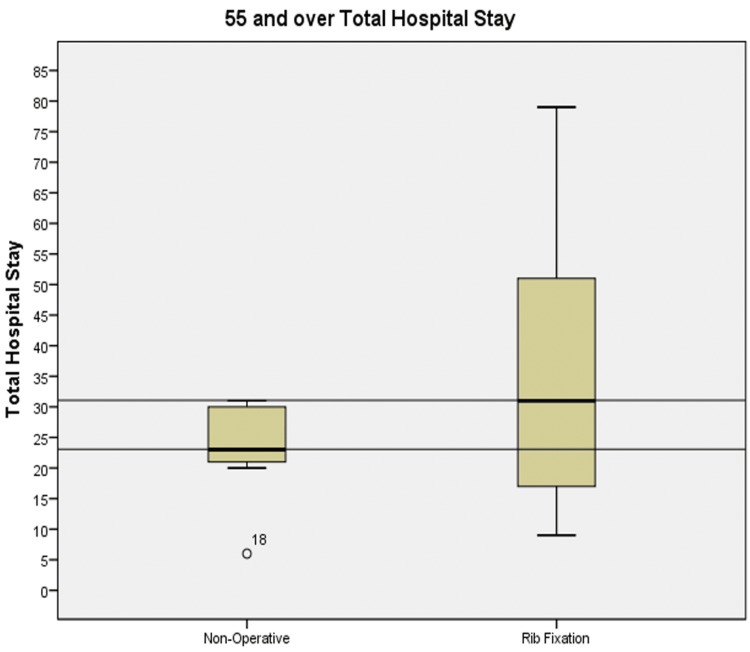
Box plots showing the total length of stay in the non-operatively managed and rib fixation cohorts for patients ≥55 years old

In the over 55 set, for the same reason as mentioned above, for the value of 753 days, we have excluded the patient who had a total length of stay of 193 days from the rib fixation group. In Figure 2, there has been an overall increase in total LOS from the non-operative group compared to the rib fixation group. There was a mean increase from 30.45 to 32.47 days with median increase from 23 to 31 days, respectively.

**Figure 3:**
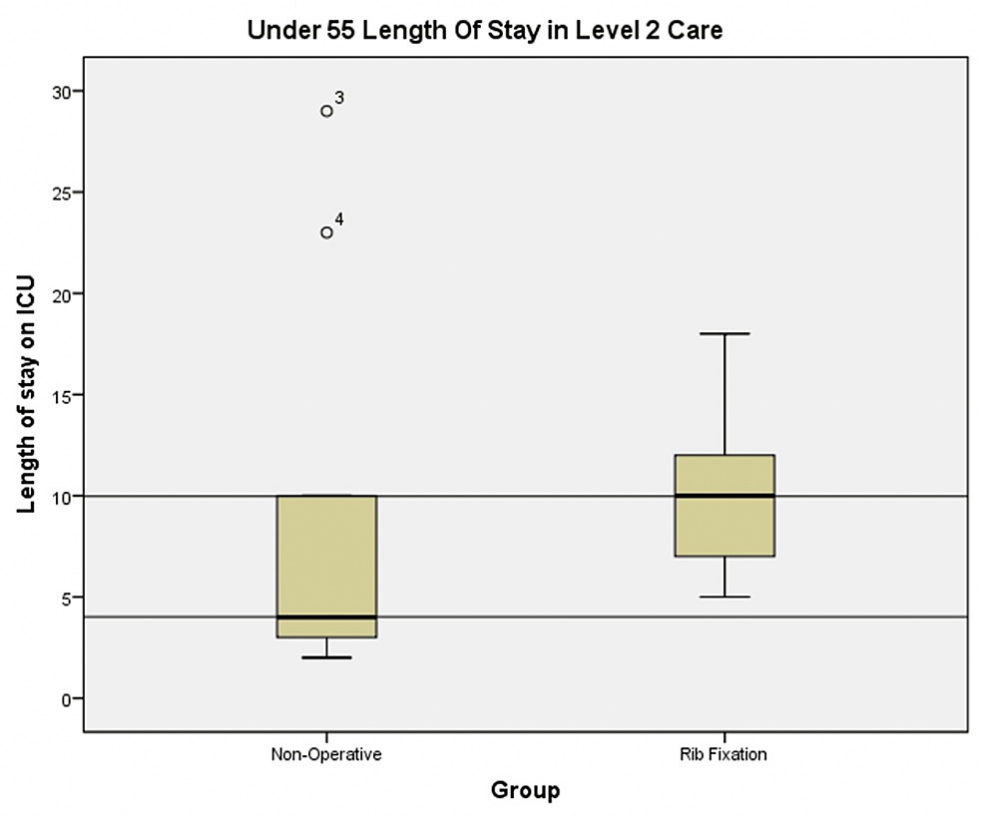
Box plots showing ICU length of stay in the non-operatively managed and rib fixation cohorts for all patients <55 years old ICU: intensive care unit

Overall, there was an increase in the length of stay in level 2 care for all patients in the operative data set. The median intensive care unit (ICU) LOS increased by six days in the under 55 group as shown in Figure 3, with an increase in mean ICU LOS stay from 9.33 to 10.18 days in the non-operative group compared to the rib fixation group. 

**Figure 4:**
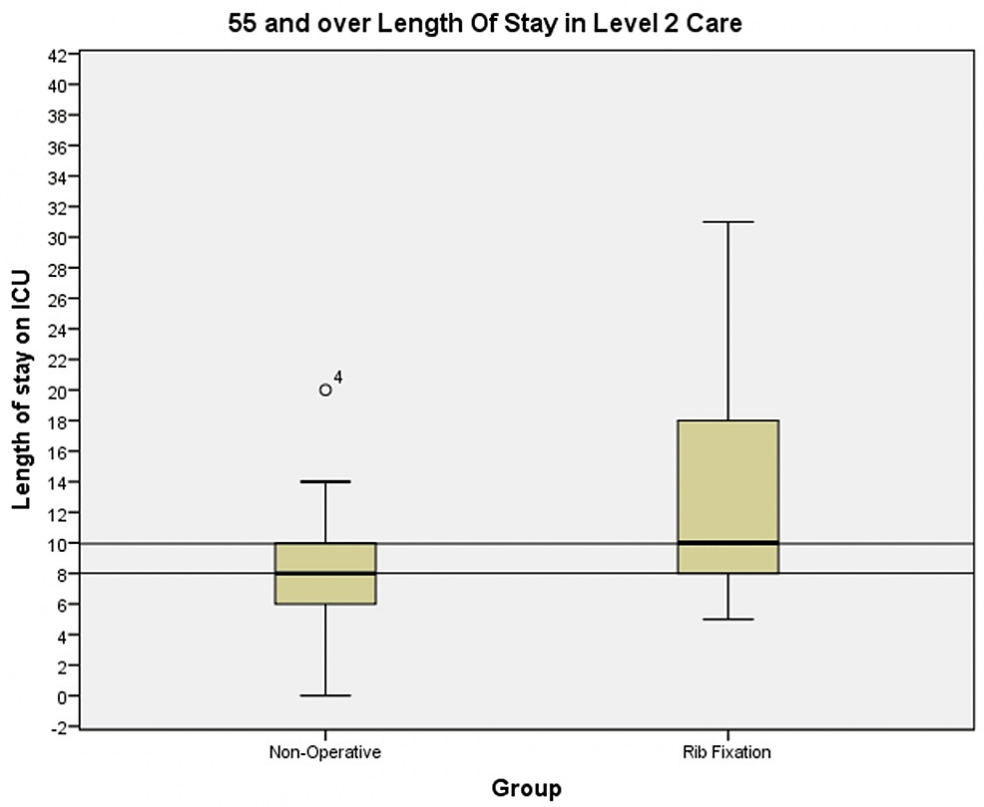
Box plots showing ICU length of stay in the non-operatively managed and rib fixation cohorts for patients ≥55 years old ICU: intensive care unit

Similarly, we observed a median increase of two days in the 55 and over category as shown in Figure 4 and a mean increase from 9.11 to 13 days in the non-operative group compared to the rib fixation group.

No statistically significant differences were found in the total hospital LOS and ICU LOS between the non-operative and rib fixation cohorts in either groups.

Service evaluation (2018)

The 2017 study (between 1 September 2015 and 27 March 2017) and the 2018 study (between 28 March 2017 and 25 May 2018) identified a total of 32 patients and 18 patients who underwent rib fixation at SRFT, respectively. The two cohorts of patients were combined and divided into the under 55 and over 55 groups, and the analysis of patient demographics and clinical characteristics for these cohorts are presented in Table 4.

**Table 4:** Patient demographics and clinical characteristics of the under 55 cohort, the over 55 cohort and the combined data of these two cohorts

Demographics/Clinical Characteristic	Under 55		Over 55		Combined Cohort	
	n = 22	%	n = 28	%	n = 50	%
Mean age (years)	42.3	-	69.64	-	57.6	-
Sex						
Male	15	68	16	57	31	62
Female	7	32	12	43	19	38
Co-morbidities						
Minor	15	68	7	25	28	56
Major	7	32	21	75	22	44
Mechanism of injury						
Blunt trauma	1	5	1	4	2	4
Fall of <2 m	1	5	8	28	9	18
Fall of >2 m	5	23	12	43	17	34
Pedestrian versus vehicle	8	36	6	21	14	28
Vehicle versus vehicle	7	32	1	4	8	16
Mean number of fractures	9.8	-	8.3	-	8.9	-
Mean number of ribs fixed	5.3	-	5.1	-	5.2	-

The under 55 group consisted of 15 males (68%) and seven females (32%) with a mean age of 42.3 years (range: 22-54). Minor co-morbidities were seen in 15 of the 22 patients (68%), and the remaining seven patients (32%) had major co-morbidities. In the over 55 group, 16 male (57%) and 12 female patients (43%) were identified with an average age of 69.6 years (range: 55-89). Major co-morbidities were more common in this group, with 21 of 28 patients (75%) having at least one major co-morbidity.

When analysing the mechanisms of injury in the two groups, injury in the under 55 group most commonly resulted from high-energy trauma, occurring in 15 of 22 (68%) patients (pedestrian versus vehicle = 36%; vehicle versus vehicle = 32%). In the over 55 group, the most common mechanism of injury was as a consequence of a fall in 20 of 28 (71%) patients (fall of <2 m = 28%; fall of >2 m = 43%). Blunt trauma was a cause of injury in one patient in both groups, representing 5% and 4%, respectively.

The number of ribs that were fractured in both groups of patients was very similar. An average of 9.8 ribs was fractured in the under 55 group (range: 5-16) compared to a mean of 8.3 rib fractures in the over 55 group (range: 5-15). Similarly, the number of ribs that were fixed was almost identical, with an average of 5.3 ribs fixed in the under 55 group (range: 3-12) and 5.1 in the over 55 group (range: 3-8).

The analysis of the data for the combined cohort shows that SRFT is operating on these patients on an average of 3.4 days following attendance to the ED. This is associated with an average of 11.1-day critical care LOS and a mean overall hospital LOS of 26.1 days.

The original study data (between 1 September 2015 and 27 March 2017) had an average of 4.1 days from ED to operation (range: 1-12), 12.7-day critical care LOS (range: 5-40) and 29.1-day overall hospital LOS (range: 9-98). The mortality rate was 3.3%, representing one patient death.

The results of the current study group revealed an improvement in all outcomes. The length of time from ED to rib fixation was reduced by 2.0 days, from an average of 4.1 days to 2.1 days (range: 0-5 days), in this study. Critical care LOS was reduced by 5.2 days, from 12.7 days to 7.5 days (range: 0-45). Likewise, the overall hospital LOS was reduced by 8.4 days, from 29.1 days to 20.7 days (range: 4-77). Both critical care LOS and overall hospital LOS are based on 17 patients as one patient was still undergoing care as an inpatient during this study. The analysis of this data is shown in Table 5.

**Table 5:** Outcome data of the original study (2017), current study (service evaluation, 2018) and a combination of the study periods ED, emergency department; LOS, length of stay

Outcome (Average Days)	Original Study	Current Study	Combined Cohort
	n = 32	n = 18	n = 50
Time from ED to rib fixation	4.1	2.1	3.4
Critical care LOS	12.7	7.5	11.1
Overall hospital LOS	29.1	20.7	26.1

This service evaluation showed an absence of mortality, which is a reduction in the number of deaths from the one death reported in the original study. Furthermore, there was an absence of complications associated with fixation, such as wound infections, pulmonary sequelae or the removal of fixation materials.

A comparison of outcomes between the separate age groups of patients (the under 55 and over 55 groups) across the two studies reveals a very similar picture, also showing a reduction in outcomes. The data comparing ED to rib fixation and regarding critical care LOS and overall hospital LOS is shown in Table 6.

**Table 6:** Outcome data comparing the under and over 55 groups between the two study periods (original study, 2017; current study, 2018 service evaluation) ED, emergency department; LOS: length of stay

Outcome (Average Days)	Under 55		Over 55	
	Original study	Current study	Original study	Current study
	n = 13	n = 9	n = 19	n = 9
Time from ED to rib fixation	3.7	1.6	4.3	2.7
Critical care LOS	10.2	4.9	14.4	10.5
Overall hospital LOS	24.4	15.6	32.4	26.5

## Discussion

MTCs across England provide a rib fixation service, and there is an international belief that it is beneficial in certain circumstances. The results published from three RCTs within the last 15 years have shown surgical management to be beneficial in patients who incur traumatic rib fractures [3]. The initial matched cohort study of the SRFT rib fixation service has shown a reduction in mortality of 33.4% when compared to independently matched patients who underwent conservative management. Further published studies have demonstrated a vast improvement in short-term outcomes, including overall length of stay in the ICU and hospital [7,19].

When comparing outcomes, the outcomes of the most recent study showed that patients who would benefit from chest stabilisation are on average being identified and undergoing rib fixation sooner. In terms of overall critical care and hospital LOS, our results showed a similar outcome to that of published studies looking at comparable outcomes [7,19]. This was also shown to be the case when comparing the under 55 and over 55 groups of the matched cohort study and service evaluation. The reduction in all outcomes in both the under 55 and over 55 groups clearly supports the surgical management of rib fractures in both the elderly and young patients.

The figures show that SRFT is fixing rib fractures earlier in all age groups. However, it is thought that elderly patients would particularly benefit from earlier fixation as they have poor physiological reserve and are more likely to suffer the sequelae associated with rib fractures. Perhaps, elderly patients with multiple rib fractures should be fixed at the earliest opportunity, which is contrary to the RTC recommendations [7] of waiting until the evidence of deterioration in respiratory function.

It is not yet known exactly how many ribs need to be stabilised in order to produce a stable chest wall. However, the results of both surgical groups lean towards fixing only a certain percentage of ribs that have been fractured. Across 50 patients, an average of 5.2 ribs was fixed per patient out of an average of 8.9 fractures per patient, and the outcomes were positive with only one death following fixation. It must also be noted that this death occurred in the initial 2017 study group 28 days following rib fixation. Equally, the number of ribs that were fixed in the under 55 and over 55 groups was almost identical at 5.1 and 5.2 ribs per patient, respectively.

Despite the exciting results, a major limitation of the service evaluation was the small sample size of only 50 patients available to produce a comparison study. Similarly, the RCTs [7] that have influenced the current NICE guideline [4] are also relatively small with 123 patients identified from three RCTs. To credibly estimate the proportion of rib fractures that require fixation to produce a stable, mechanically functional chest wall, a larger study is necessary. In order to be able to appropriately determine the efficacy of rib fixation, a multicentre prospective randomised control trial is needed with standardised indications for rib fracture fixation in all patients with both flail and non-flail chest injuries.

## Conclusions

The SRFT initial study of rib fracture fixation in patients with multiple rib fractures revealed a lower LOS in young patients and a significant reduction in mortality in the elderly. The service evaluation identifies that the SRFT Chest Injuries Group is identifying patients for fixation sooner with an average time to fixation having reduced from 4.1 days to 2.1 days. This could be explained as with every new service, there is a learning curve, and the clinicians involved are becoming more experienced in the management of patients with multiple rib fractures. The LOS has further decreased in the second study, which could be explained by earlier fixation results in the avoidance of the sequelae related to rib fractures, and therefore, the patients have a better outcome more quickly. However, whilst SRFT has clearly improved on their outcomes between the first and second studies, there is still work to be done not only on the optimal time for fixation but also on how many or which ribs should be fixed for an optimal outcome.
